# Role of methotrexate chronotherapy in collagen-induced rheumatoid arthritis in rats

**DOI:** 10.1007/s00393-016-0236-6

**Published:** 2016-11-29

**Authors:** X. Wang, X. Yan, F. Wang, F. Ge, Z. Li

**Affiliations:** Binzhou medical university hospital, Binzhou city, Shandong province China

**Keywords:** Cytokine, Circadian rhythm, Inflammatory, Treatment, Histopathyology, Cytokin, Biorhythmus, Entzündung, Behandlung, Histopathologie

## Abstract

**Aim:**

To explore the circadian rhythm of serum interleukin (IL)-6 in collagen-induced arthritis (CIA) rats and compare the safety and effectiveness of methotrexate (MTX) administered traditionally and via chronotherapy.

**Methods:**

CIA rat models were immunized with bovine type II collagen. Serum IL-6 levels in normal and CIA rats were measured at 2, 6, 10, 14, 18, or 22 h after the light was turned on (HALO). MTX was administered to 6 HALO/18 HALO experimental groups of Wistar rats once daily according to the IL-6 rhythm. The control groups (positive, negative, and normal) were given MTX or an equal volume of phosphate buffered saline (PBS) once a week simultaneously. Arthritis score, tumor necrosis factor (TNF)-α, interleukin (IL)-6, and C reactive protein (CRP) levels in the serum were measured by enzyme-linked immunosorbent assay (ELISA). Histological changes in the ankle joint were analyzed.

**Results:**

After 6 weeks of treatment, arthritis scores in the experimental group were lower than in the control group. The expression of TNF-α, IL-6, and CRP was lower in the 18 HALO group than in the control or 6 HALO groups. Histopathology scores in the experimental groups were lower than in the control group (*p* < 0.05).

**Conclusion:**

The plasma IL-6 levels in CIA rats were higher than in normal rats and showed significant circadian rhythm. Daily administration of MTX is more potent than weekly administration. The therapeutic index of rheumatoid arthritis (RA) may be improved with MTX therapy based on the IL-6 circadian rhythm.

## Zusammenfassung

**Ziel der Arbeit:**

Ziel war es, den zirkadianen Rhythmus von Interleukin-6 (IL-6) im Serum von Ratten mit kollageninduzierter Arthritis (CIA) zu untersuchen sowie die Sicherheit und Wirksamkeit von Methotrexat (MTX) bei herkömmlicher Gabe und per Chronotherapie zu vergleichen.

**Methoden:**

Als Modell der CI dienende Ratten wurden mit Kollagen vom bovinen Typ II immunisiert. Die Serumspiegel von IL-6 wurden bei normalen und CIA-Ratten 2, 6, 10, 14, 18 oder 22 h nach Einschalten des Lichts (HALO) gemessen. Den Wistar-Ratten in den Versuchsgruppen 6 HALO/18 HALO wurde einmal täglich MTX entsprechend dem IL-6-Rhythmus verabreicht. Gleichzeitig wurde den Kontrollgruppen (positiv, negativ und normal) einmal pro Woche MTX oder das gleiche Volumen phosphatgepufferter Salzlösung („phosphate buffered saline“, PBS) gegeben. Ermittelt wurden der Arthritis-Score sowie die Serumspiegel von Tumornekrosefaktor-alpha (TNF-α), IL-6 und C‑reaktivem Protein (CRP) anhand der Messung mittels ELISA-Test („enzyme-linked immunosorbent assay“). Außerdem wurden histologische Veränderungen im Fußgelenk untersucht.

**Ergebnisse:**

Nach 6‑wöchiger Therapie waren die Arthritis-Scores in der Versuchsgruppe niedriger als in der Kontrollgruppe. Die Expression von TNF-α, IL-6 und CRP war in der Gruppe 18 HALO geringer ausgeprägt als in der Kontrollgruppe und der Gruppe 6 HALO. Darüber hinaus waren die histopathologischen Werte in den Versuchsgruppen niedriger als in der Kontrollgruppe (*p* < 0,05).

**Schlussfolgerung:**

Die Werte für IL-6 im Plasma waren bei CIA-Ratten höher als bei normalen Ratten und wiesen einen signifikant ausgeprägten zirkadianen Rhythmus auf. Dabei stellte sich die tägliche Gabe von MTX als wirksamer heraus denn die wöchentliche Verabreichung. Der therapeutische Index bei rheumatoider Arthritis (RA) lässt sich möglicherweise mit einer MTX-Therapie verbessern, die auf dem zirkadianen Rhythmus von IL-6 basiert.

## Introduction

Rheumatoid arthritis (RA) is an autoimmune disorder of unknown etiology. In industrialized countries, rheumatoid arthritis affects 0.5–1.0% of adults, with 5–50 new cases per 100,000 annually [1]. RA not only induces disability that affects physical function, but also results in premature death. Patients with RA have a poor life prognosis and a substantially shorter life expectancy compared to the general population [2]. Therefore, the development of an effective therapeutic strategy focusing on prevention and treatment of the disease, its complications, and associated disorders is imperative.

Methotrexate (MTX), which has been used for more than 40 years in the treatment of rheumatoid arthritis, has an anti-inflammatory cytotoxic and immunosuppressive effect. Currently, a small dose of intermittent medication is the standard treatment for RA, with proven efficacy and safety. The therapeutic mechanisms of MTX in RA have yet to be fully elucidated. The plasma half-life of MTX was 7 to 10 h, onset time 6 to 8 weeks, and the optimal therapeutic effect was seen after 2 to 3 months [3, 4]. MTX administrated twice weekly, according to the half-life of MTX, was better than once weekly. However, the pilot study of Pandya has confirmed that the benefit was similar in either approach [5]. An uncontrolled trial conducted by To et al. suggested that MTX chronotherapy can improve RA symptoms compared to the current standard dosing methods [14]. Therefore, current clinical approaches involve oral or intramuscular injection of MTX once weekly and 12 h of continuous therapy twice or three times a week. The first approach is widely accepted, owing to high patient compliance and flexibility.

In recent years, inflammatory cytokines such as tumor necrosis factor-α (TNF-α) and interleukin 6 (IL-6) have been observed in the blood and synovial fluid of humans, with excessive production of these cytokines implicated in the pathogenesis of RA [6, 7]. The levels of inflammatory cytokines increased in the early morning in RA patients, with a clear circadian rhythm in the blood [8–10]. Chronotherapy is defined as the administration of medication based on biological rhythms in order to optimize therapeutic outcomes and/or control adverse effects. RA chronotherapy has been researched basically and clinically with glucocorticoids [11, 12], MTX [13, 14], tacrolimus [15], and mizoribine [16, 17]. The results showed that selection of the optimal dosage associated with a circadian rhythm of RA symptoms resulted in an effective treatment for RA.

In view of these findings, we decided to investigate methotrexate chronotherapy using a collagen-induced arthritis (CIA) rat model immunized with bovine type II collagen. In this study, serum IL-6 levels were measured at six different time points. MTX was administered at two different dosing intervals based on these findings, and its efficacy and toxicity were evaluated.

## Materials and methods

### Animals

Six-week-old, female Wister rats weighing 160 to 170 g each were purchased from the Experimental Animal Center of Shandong (Shandong, China). The study was conducted at Bin Zhou (Shandong, China). They were housed 6 in each cage under standardized light-dark cycle conditions (lights-on and lights-off at 07:00 and 19:00 h, respectively) at a room temperature of 24 ± 1 °C and a humidity of 60 ± 10% with free access to food and water. Experiments were conducted after formal approval by the Institutional Ethical Committee for Research on Animals.

### Reagents

The reagents included bovine collagen type II (CII) and complete Freund’s adjuvant (CFA; Chondrex, Washington, USA), as well as MTX (Shanghai Sine Pharmaceutical Co., Ltd., Shanghai, China). Enzyme-linked immunosorbent assay (ELISA) kits of TNF-α, IL-6, and CRP (R&D system, Minnesota, USA) were purchased. All the other chemicals and biochemicals used were of the highest grade available.

### Induction of collagen-induced arthritis

Bovine collagen type II (CII) was dissolved in 0.05 mol/L acetic acid at 2 mg/ml and emulsified in an equal volume of CFA. Rats were immunized intracutaneously with 200 µl of the emulsion divided into four points along the dorsal spine symmetrically. Seven days later, a similar amount of bovine CII emulsified into CFA was injected intracutaneously as a booster dose.

### Evaluation of arthritis

The severity of CIA was assessed using a scoring system [18]. The disease severity was recorded for each limb as follows: score 0, normal; 1, erythema and mild swelling confined to the midfoot (tarsals) or ankle joint; 2, erythema and mild swelling extending from the ankle to the midfoot; 3, erythema and moderate swelling extending from the ankle to the metatarsal joint; and 4, erythema and severe swelling of the ankle, foot, and digits. The clinical arthritis score was defined as the sum of the scores of all four paws of each rat. Evaluation was completed by two experimenters independently. Final score included the average of the two scores. Arthritis index (AI) represented the total joint score. The model was successful when the AI was equal to or greater than 4.

### Groups and treatment

Wistar rats were divided randomly into five groups (*n* = 8 per group): the 6 hours after the light was turned on (HALO) and the 18 HALO experimental groups, the qw positive controls, the PBS negative controls, and the normal control group. MTX (1/7 mg/kg) was administered at 6 HALO when the IL-6 level started to decrease and at 18 HALO when the IL-6 level started to increase, once daily by gastric perfusion, separately from day 1 to day 56 after successful modeling. In the qw (once a week) positive control group, MTX (1 mg/kg) was administered once weekly by gastric perfusion. The PBS control and the normal groups were given an equal volume of phosphate buffer saline (PBS) simultaneously.

### IL-6 level after initial immunization

IL-6 levels of the rats were measured on days 0, 7, 14, 21, 28, and 35 for signs of arthritis between days 1 and 36 after initial immunization. Blood was drawn from the caudal vein of CIA rats (*n* = 5) on days 0, 7, 14, 21, 28, and 35 after initial immunization. Serum was obtained by centrifugation at 3000 rev/min for 10 min and frozen at −80 °C until assay. We measured IL-6 levels using an ELISA kit.

### 24-hour rhythm in serum IL-6 levels

On day 21 after immunization, blood was collected at six different times (2, 6, 10, 14, 18, or 22 HALO) in normal (*n* = 5) or untreated CIA (*n* = 8) rats, and centrifuged immediately at 3000 rpm for 10 min. Serum was obtained and stored at −20 °C until analyzed.

### Influence of MTX chronotherapy on arthritis score

The rats were assessed every 3 days for signs of arthritis between days 1 and 56 after immunization using the scoring system [18].

### Measurement of serum TNF-α, IL-6, and CRP level

Blood was collected and serum was obtained by centrifugation at 3000 rpm for 10 min and stored at −80 °C prior to analysis. Serums IL-6 levels were quantified by ELISA. The same procedure was used to assay TNF-α and CRP with the cytokine-specific antibodies.

### Histological analysis

On day 56, all rats were sacrificed via anesthesia after serum collection. The ankle joints were removed from the rats for histological examination. The joints were fixed in 10% phosphate-buffered formalin, decalcified in 10% EDTA for 30 days at 4 °C, and then embedded in paraffin. Serial paraffin sections (5 mm thick) were stained with hematoxylin and eosin (H&E). Pathological sections were examined and graded for inflammation, pannus formation, cartilage damage, and bone resorption microscopically, and scored using histopathology scoring criteria [19].

### Leukocyte count analysis

On day 56, blood samples were drawn from the inferior vena cava and leukocyte counts of the rats were performed immediately after blood drawing.

### Statistical analysis

The results were analyzed using a statistical program SPSS/PC+, version 13.0 (SPSS Inc, Chicago, IL, USA). One-way analysis of variance (ANOVA) and repeated-measures ANOVA were used to determine the statistically significant differences between the various experimental groups. Differences between groups were determined using Scheffe’s or Fisher’s protected least significant difference test. Data are expressed as means ± standard deviation (SD) and *p* < 0.05 was considered statistically significant.

## Results

### Increased IL-6 levels after immunization and circadian rhythm of serum IL-6 levels

**Fig. 1:**
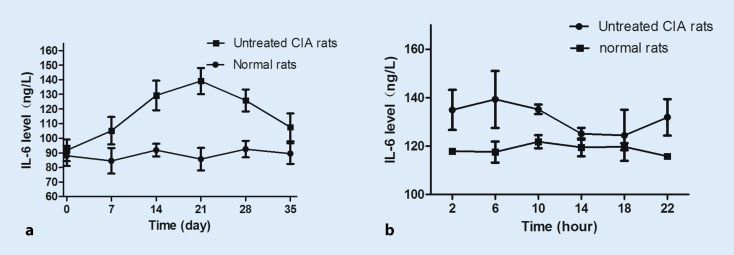
Interleukin 6 (*IL-6*) levels of rats after initial immunization. The IL-6 levels in collagen-induced arthritis (*CIA*) rats increased after immunization and peaked on day 21 (**a**). Circadian rhythms of plasma IL-6 in normal (*n* = 5) and CIA (*n* = 8) rats (**b**). Each value represents the mean ± standard deviation. (*p < 0.05 compared with the normal group)

The IL-6 levels started to increase after immunization and peaked on day 21; they then decreased and eventually returned to normal levels on day 35 (Fig. 1a). Serum IL-6 concentrations in collagen-induced arthritic rats were higher than in normal rats on day 21. IL-6 levels showed significant circadian rhythms, with higher levels in 6 HALO and lower levels in 18 HALO CIA rats (Fig. 1b). Serum IL-6 showed no circadian rhythm in normal rats (*p > *0.05).

### Effect of MTX chronotherapy on arthritis score and pathology changes

**Fig. 2:**
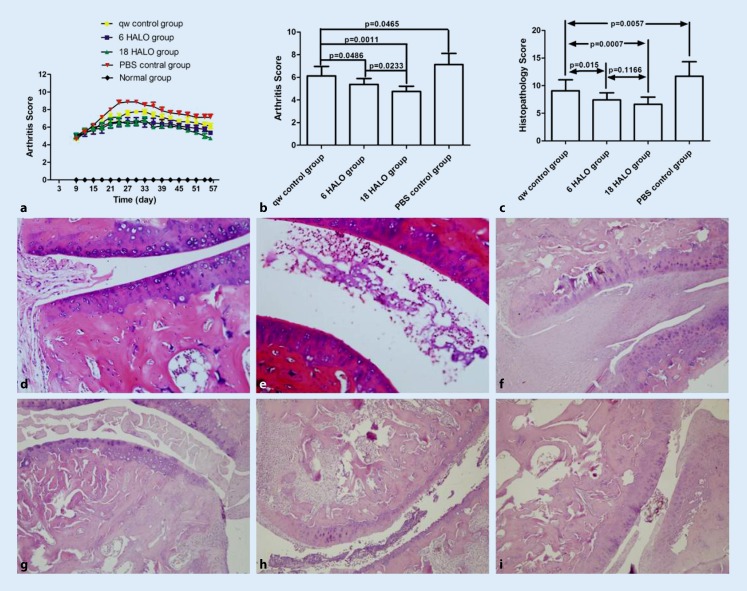
Influence of methotrexate chronotherapy on arthritis score and ankle joint histopathological changes. Data represent the mean ± standard deviation (*n* = 8). The arthritis scores were assessed in each group (**a**) and suppressed peak arthritic severity compared with the once a week (qw) and blank control group was seen on day 56 (**b**). **c** Histopathology score comparisons between groups on day 56. Histopathology: **d** normal group, **e** collagen-induced arthritis rats, **f** phosphate-buffered saline (*PBS*) control group, **g** qw control group, **h** 6 hours after the light was turned on (HALO) experimental group, **i** 18 HALO experimental group

In the study, the arthritis scores were assessed in each group, which each included 8 rats. In the normal group, there was no joint swelling during the whole experiment and the arthritis score was zero at each time point. Rats on days 7–10 after initial immunization showed congestion and edema of ankle joints. On days 10 to 16 after initial immunization, the ankles showed swelling and hyperemia, which increased by day 21, followed by a gradual decline in joint swelling (Fig. 2a). MTX administrated once weekly or once daily decreased the arthritis score significantly (Fig. 2a). On day 56, the arthritic severity in the 6 HALO/18 HALO experimental groups was markedly decreased compared to the qw and PBS control groups on day 56 (*p* < 0.05). The Arthritis score in the 18 HALO experimental group was obviously decreased compared to the 6 HALO experimental group (*p < *0.05; Fig. 2b).

### Effect of MTX dosing time on pathology changes

After MTX treatment, histopathology scores were reduced (*p* < 0.05) and the histopathology scores were lower in 6 am and 18 HALO experimental groups than in the qw positive control group (*p* < 0.05; Fig. 2c). The effect of MTX dosing time on ankle joint histopathological changes varied. The normal group of rats showed normal articular cartilage and absence of damage in the synovium (Fig. 2d). The CIA rats showed marked infiltration of inflammatory cells (Fig. 2e). The PBS control group showed marked infiltration of inflammatory cells and synovial hyperplasia, and the mean histopathology score was 11.7 ± 2.6 (Fig. 2f). The qw positive control group was treated with MTX once a week and the mean histopathology scores were 9.07 ± 1.98 (Fig. 2g). The mean histopathology score in the 6 HALO experimental group was 7.43 ± 1.28 (Fig. 2h) while this was 6.64 ± 1.27 in the 18 HALO experimental group (Fig. 2i).

### Effect of MTX dosing time on TNF-α, IL-6, and CRP levels

**Fig. 3:**
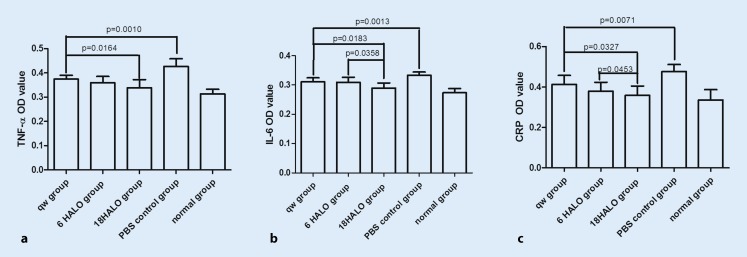
Effect of methotrexate dosing time on tumor necrosis factor-α (TNF-α), interleukin (IL-6), and C reactive protein (CRP) levels. The (optical density) OD value of TNF-α, IL-6, and CRP in the 18 hours after the light was turned on (HALO) experimental group was markedly decreased compared with the phosphate-buffered saline (*PBS*) and once a week (qw) control groups (*p* < 0.05; **a–c**). The OD values of IL-6 (**b**) and CRP (**c** ) in the 18 HALO group were markedly decreased compared with those of the 6 HALO group

MTX was administered every day at 6 HALO or 18 HALO based on the circadian rhythm of IL-6 level. The OD values of TNF-α, IL-6, and CRP in the 18 HALO experimental group were markedly decreased compared with the PBS and qw control groups (*p* < 0.05; Fig. 3). The OD values of IL-6 (Fig. 3b) and CRP (Fig. 3c) in the 18 HALO group were markedly decreased compared with those of the 6 HALO group.

### Effect of MTX dosing time on myelosuppression

**Fig. 4:**
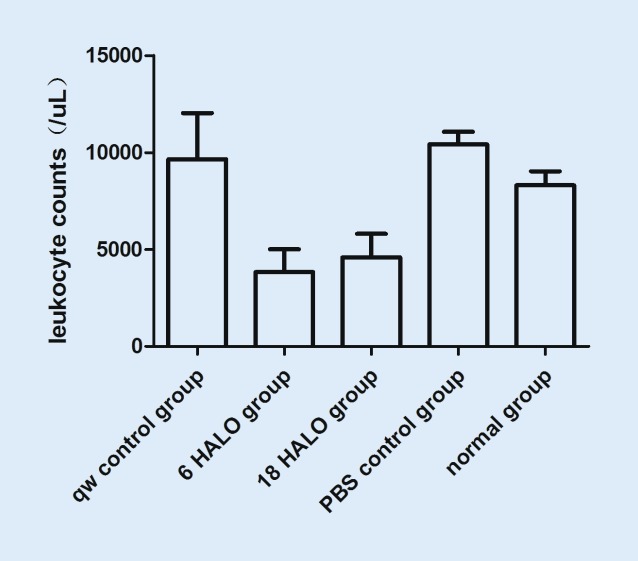
Effect of methotrexate dosing time on leukocyte counts. On day 56, blood samples were drawn from the inferior vena cava and leukocyte counts of the rats were performed immediately after blood drawing. Values represent the mean ± standard deviation. Leukocyte counts were lower in 6 hours after the light was turned on (HALO) and 18 HALO experimental groups than in controls (*p* < 0.05), but are still within the normal range. *PBS* phosphate-buffered saline, *qw* once a week

After MTX treatment, leukocyte counts were lower in the 6 HALO and 18 HALO experimental groups than in controls (Fig. 4, *p* < 0.05), but they were still within the normal range.

## Discussion

Rheumatoid arthritis (RA) is a chronic progressive inflammatory disease with massive infiltration of inflammatory cells in the synovium of multiple joints. Monocytes, T and B lymphocytes, and fibroblasts produce inflammatory cytokines including IL-6, TNF, and other proinflammatory cytokines. These cytokines contribute to RA pathogenesis and were reduced rhythmically corresponding to major symptoms, such as pain, inflammation, and stiffness [12, 20, 21].

The CIA rat model mimics certain features of RA [22]. It is internationally recognized for the study of RA. In our study, we have measured IL-6 levels (Fig. 1a) of rats after immunization. We found that the IL-6 level was relatively high on day 21. Therefore, in a later study, we measured the levels of IL-6 at six different time points on day 21. We found that the level of IL-6 showed a clear 24-hour rhythm, with higher levels at 6 HALO and lower levels at 18 HALO in the CIA rats (Fig. 1b).

RA is characterized by synovial hyperplasia, pannus formation, and cartilage and bone destruction in the joint. In our study, we found that 1 week after immunization, the skin of the foot joints in rats was flushed, with mild swelling. After 2 weeks, most of the rat ankle joint was swollen, with shiny skin and limited joint mobility. After 3 weeks, skin ulcers were found in some rats, and joint swelling and deformation were found after 5 to 6 weeks. In our study, we assessed the arthritis score of the rats (Fig. 2a) and compared the arthritis index of each group on day 56 (Fig. 2b). At the end of the experiment, the rats were sacrificed and the ankle joints were removed for histological examination and histopathological evaluation by H&E staining. The ankle joints, cartilage, and joint space in normal rats were normal. The knee joints of the CIA rats displayed notable synovial hyperplasia, inflammatory cell filtration, and partial bone destruction (Fig. 2d–i). The scores of the experimental groups were significantly lower than that of the control group (Fig. 2c). Studies have shown that treatment based on circadian rhythms might delay the progression of pathology.

TNF-α plays an important role in the pathogenesis of RA. It mediates inflammatory cell infiltration, cartilage destruction, and systemic inflammation. It also induces the synthesis of proinflammatory cytokines (such as IL-1 and IL-6) and chemokines, and activates macrophages, which in turn perpetuate the inflammation in autoimmune pathology [23, 24]. Plasma TNF-α and TNF-α mRNA expression showed a clear circadian rhythm, with peaks 2 h after the light was turned on in MRL/lpr mice, leading to an excellent outcome when MTX was administered under increased cytokine conditions [13, 14]. Some researchers have postulated that MTX reduces plasma TNF-α level by suppressing transcriptional activity rather than by suppressing lymphocyte proliferation [14]. In our study, the levels of plasma TNF-α were measured. We found that the value of TNF-α in the 18 HALO experimental group was markedly decreased compared with the PBS and qw control groups (*p* < 0.05; Fig. 3a). These results indicate that MTX once daily is superior to once weekly.

IL-6 is a key proinflammatory cytokine in RA. It mediates osteoclast activation and fibroblast synovial cell activation, thus contributing to synovial pannus formation, joint destruction, and bone and cartilage breakdown. In our study, we demonstrated higher plasma IL-6 levels in CIA rats than in normal rats at almost every sampling time, with a significant circadian rhythm indicating higher levels during the light phase and lower levels in the dark phase (Fig. 1b). Based on the circadian rhythm, we administered MTX once daily according to a chronotherapy schedule, in which MTX was given at 6 HALO or 18 HALO without altering the dosage. We assessed arthritis score; production of TNF-α, IL-6, and CRP in serum; and histological changes of ankle joints. In rats, the arthritis score was significantly lower in the 18 HALO experimental group than in the control and 6 HALO experimental groups on day 24. On day 56, the 18 HALO experimental group treatment significantly inhibited the increase of arthritis score compared to the control and 6 HALO experimental groups in rats (Fig. 3b), and showed a clear improvement in inflammatory cytokines. These results indicate that MTX administered once daily was better than once weekly, and that MTX chronotherapy was effective in treating CIA rats.

CRP is a marker of inflammatory responses. Plasma CRP levels showed a circadian rhythm, with a peak in the early morning in RA patients that matched the rhythms of pain and stiffness [25]. Studies have confirmed that CRP levels showed a clear circadian rhythm with a peak at 22 HALO, in accordance with those seen in CIA and MRL/lpr mice [13–15]. In our study, after 8 weeks of treatment, the CRP level in the 18 HALO experimental group showed a clear decrease compared to the qw group (Fig. 3c). Our study suggests that MTX once daily has an advantage over once weekly in terms of efficacy, and thus the currently used once-weekly regimen is good enough until alternate dosing schedules are evaluated in a large double-blind, randomized, controlled multicentric trial.

Leukocyte counts increase [26, 27] in most RA patients and induce an inflammatory response. RA symptoms markedly improved following leukocytapheresis [28]. Leukocyte counts were measured in MRL/lpr mice and were found to be significantly increased after RA onset. Circadian variations in the toxicity of MTX have also been demonstrated in rats, with maximum toxicity occurring after dosage at 06:00 h and minimum toxicity after dosage at midnight. Administration at the other two time points, 12.00 h and 18.00 h, gave intermediate results [29]. A common side effect of MTX is leucopenia in RA patients. In this study, we found that leukocyte counts decreased in 6 HALO and 18 HALO experimental groups, but increased in the qw positive control group (Fig. 4). Inhibition of increased leukocyte counts may be one of the options available for treatment of RA. In the current study, we also found a slow increase in body weight of rats following treatment, which may account for the adverse gastrointestinal effects of MTX.

The role of proinflammatory cytokines, including TNF-α and IL-6, in the pathogenesis of RA has led to the development of biological disease modifying antirheumatic drugs (bDMARDs), which specifically inhibit these cytokines. Selecting the optimal therapeutic dosage based on circadian rhythms of inflammation and cytokine response in RA may result in successful outcomes.

It has been reported in previous studies that CIA mice administered MTX in the resting phase showed a larger area under the plasma concentration–time curve of MTX than mice administered MTX in the active phase [13], and that the daily variations in the pharmacokinetics of MTX were not related to the dosing-time dependency of MTX efficacy [14]. From the results of our studies and the 24-hour cycles of cytokines in RA patients [8, 10], it is thought that MTX has a significant dosing time-dependent anti-inflammatory action and that this effect may be due to the 24-hour rhythms of cytokine levels rather than the pharmacokinetics of MTX.

## Conclusion

This study indicated that plasma IL-6 levels in CIA rats were higher than in normal rats and showed a significant circadian rhythm. The RA therapeutic score may be improved by administering MTX once daily, according to the circadian rhythm of IL-6.
